# Comparison of Gene Regulatory Networks via Steady-State Trajectories

**DOI:** 10.1155/2007/82702

**Published:** 2007-05-22

**Authors:** Marcel Brun, Seungchan Kim, Woonjung Choi, Edward R Dougherty

**Affiliations:** 1 Computational Biology Division, Translational Genomics Research Institute, Phoenix, AZ 85004, USA; 2 School of Computing and Informatics, Ira A. Fulton School of Engineering, Arizona State University, Tempe, AZ 85287, USA; 3 Department of Mathematics and Statistics, College of Liberal Arts and Sciences, Arizona State University, Tempe, AZ 85287, USA; 4 Department of Electrical and Computer Engineering, Texas A&M University, College Station, TX 77843, USA; 5 Cancer Genomics Laboratory, Department of Pathology, University of Texas M.D. Anderson Cancer Center, Houston, TX 77030, USA

## Abstract

The modeling of genetic regulatory networks is becoming increasingly widespread in the study of biological systems. In the abstract, one would prefer quantitatively comprehensive models, such as a differential-equation model, to coarse models; however, in practice, detailed models require more accurate measurements for inference and more computational power to analyze than coarse-scale models. It is crucial to address the issue of model complexity in the framework of a basic scientific paradigm: the model should be of minimal complexity to provide the necessary predictive power. Addressing this issue requires a metric by which to compare networks. This paper proposes the use of a classical measure of difference between amplitude distributions for periodic signals to compare two networks according to the differences of their trajectories in the steady state. The metric is applicable to networks with both continuous and discrete values for both time and state, and it possesses the critical property that it allows the comparison of networks of different natures. We demonstrate application of the metric by comparing a continuous-valued reference network against simplified versions obtained via quantization.

## 

[1,2,3,4,5,6,7,8,9,10,11,12,13,14,15,16,17,18,19,20,21,22,23]
